# Functions and mechanisms of protein disulfide isomerase family in cancer emergence

**DOI:** 10.1186/s13578-022-00868-6

**Published:** 2022-08-14

**Authors:** Nisa Syakila A. Rahman, Syazalina Zahari, Saiful Effendi Syafruddin, Mohd Firdaus-Raih, Teck Yew Low, M. Aiman Mohtar

**Affiliations:** 1 UKM Medical Molecular Biology Institute (UMBI), Universiti Kebangsaan Malaysia, 56000 Kuala Lumpur, Malaysia; 2 Department of Applied Physics, Faculty of Science and Technology and Institute of Systems Biology (INBIOSIS), Universiti Kebangsaan Malaysia, 43600 UKM Bangi, Selangor Malaysia

## Abstract

The endoplasmic reticulum (ER) is a multi-layered organelle that is essential for the synthesis, folding, and structural maturation of almost one-third of the cellular proteome. It houses several resident proteins for these functions including the 21 members of the protein disulfide isomerase (PDI) family. The signature of proteins belonging to this family is the presence of the thioredoxin domain which mediates the formation, and rearrangement of disulfide bonds of substrate proteins in the ER. This process is crucial not only for the proper folding of ER substrates but also for maintaining a balanced ER proteostasis. The inclusion of new PDI members with a wide variety of structural determinants, size and enzymatic activity has brought additional epitomes of how PDI functions. Notably, some of them do not carry the thioredoxin domain and others have roles outside the ER. This also reflects that PDIs may have specialized functions and their functions are not limited within the ER. Large-scale expression datasets of human clinical samples have identified that the expression of PDI members is elevated in pathophysiological states like cancer. Subsequent functional interrogations using structural, molecular, cellular, and animal models suggest that some PDI members support the survival, progression, and metastasis of several cancer types. Herein, we review recent research advances on PDIs, vis-à-vis their expression, functions, and molecular mechanisms in supporting cancer growth with special emphasis on the anterior gradient (AGR) subfamily. Last, we posit the relevance and therapeutic strategies in targeting the PDIs in cancer.

## Introduction

Approximately one-third of the cellular proteome passes through the secretory pathway before they are secreted or translocated to their destined subcellular localizations. The endoplasmic reticulum (ER) constitutes the first compartment of this secretory pathway. It orchestrates the synthesis, folding, and structural maturation of many secreted and cell surface proteins. Initially, most polypeptides enter the ER unfolded. There, with the assistance of ER-resident proteins and chaperones, they fold and mature into proper tertiary or quaternary structures, before being transported to the second compartment i.e., the Golgi-apparatus for post-translational modifications such as glycosylation and lipidation [1].

Crucial to the functions of the ER are the ER-resident proteins in the ER lumen [2]. A major component of these ER-resident proteins includes the protein disulfide isomerases (PDIs), a class of multi-domains, multi-functional enzymes that belongs to the thioredoxin superfamily. The PDI family comprises several divergent proteins that can serve as molecular chaperones for protein synthesis and maturation [3]. As a folding catalyst, the PDI proteins are capable of preserving the native conformation and stability of other proteins via the formation, isomerization and rearrangement of disulfide (S–S) bonds. The formation of S–S bonds between two cysteine residues is highly conserved, and at least 30% of all eukaryotic proteins have at least one S–S bond, and  ~ 80% of them are secreted or membrane proteins [4]. The essential roles of PDIs in oxidative protein folding are therefore conspicuous, especially the stability of the S–S bonds that can be perturbed by the reducing environments of most cellular compartments leading to protein misfolding. On the other hand, cellular functions are heavily dependent on the proper folding of proteins. To achieve proteome stability, the proteostasis network, that is comprised of conserved stress signaling pathways, involves in regulating the synthesis, folding, trafficking and degradation of proteins [5, 6]. Of note, ER proteostasis is likewise crucial because even the chronic expression of a single misfolded protein can result in the imbalance of proteostasis and impact multiple biochemical pathways [7–9]. These imbalances are associated with protein aggregation or proteinopathies which can lead to both loss- or gain-of-function diseases [10].

In this review, the structural properties and expression of PDIs in cancer are discussed. We describe existing and emerging roles as well as molecular mechanisms in supporting key biological processes in cancer emergence, with special emphasis on the anterior gradient (AGR) subfamily. In addition, the relevance and therapeutic strategies targeting the PDIs are highlighted.

## The composition and architecture of PDI family members

**Fig. 1:**
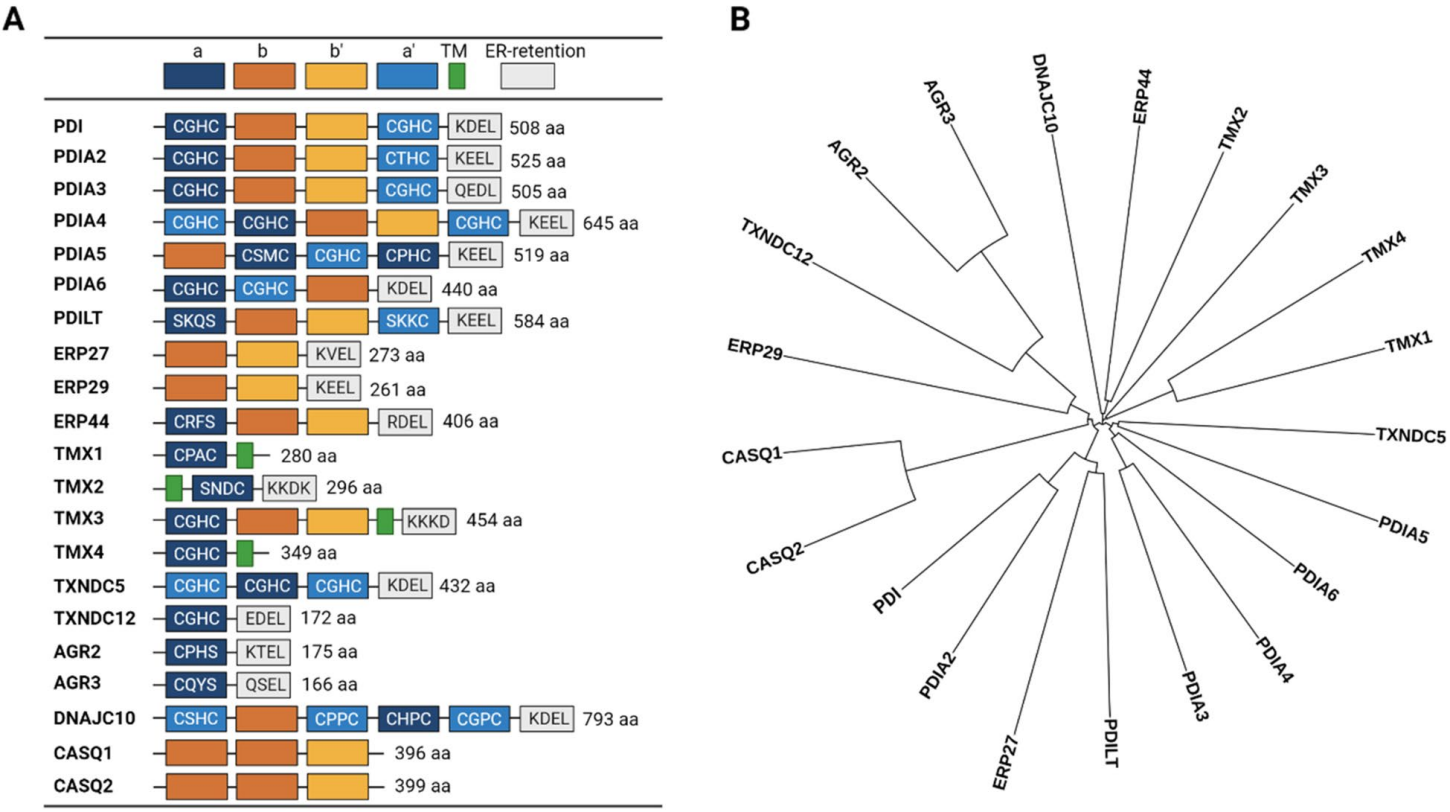
Domain representation of PDI family members and family tree. **A** Schematic representation highlighting the a- and b-type domain arrangements, transmembrane domain (TM), and ER retention motifs of all 21 PDI family members. **B** Protein sequences of all 21 PDI family members were aligned using Clustal Omega and the corresponding matrix represented as a circular tree using the iTOL website (http://itol.embl.de/)

To date, twenty-one members of the PDI protein family have been identified, each having a different size, structure, tissue distribution and enzymatic activity (Table 1) [11]. A signature of the PDI family members is the presence of a catalytic thioredoxin fold (TRX), also known as the CXXC (Cys-X-X-Cys) motif, where X can be any amino acid. However, there are proteins in this family without the catalytic TRX or non-reactive TRX. Thus, a more accurate definition of the PDI family is that it (i) contains a non-thiol-reactive TRX motif with chaperone-like folding activities as well as (ii) having sequence and structural similarity to the TRX motif. The TRX motif contains two free thiol groups at each of the cysteine residues which mediate oxidoreductase activity that is important for shuffling the S–S bonds during protein maturation [12]. Most PDI family members have one to three TRX like-motif [13] and the most conserved TRX sequence for the PDI family is CGHC [14] (Fig. 1A).

**Table 1:** The human PDI family members

Gene name	Protein name	Aliases	Uniprot ID	Chromosome	PDB code
P4HB	PDI	PDIA1, PROHB, DSI, GIT, PDI, PO4HB, P4Hbeta	P07237	17q25.3	3bj5, 4ju5, 3uem, 6i7s, 4ekz, 4el1, 1x5c, 2bjx, 1bjx, 1mek, 2k18
PDIA2	PDIA2	PDIA2, PDI, PDIR	Q13087	16p13.3	None
PDIA3	PDIA3	P58, ERp61, ERp57, ERp60, GRP57, PI-PLC, HsT17083	P30101	15q15.3	2h8l, 2alb, 3f8u, 2dmm, 6eny
PDIA4	PDIA4	ERP70, ERP72	P13667	7q36.1	3idv
PDIA5	PDIA5	PDIR, FLJ30401	Q14554	3q21.1	4i6x
PDIA6	PDIA6	P5, ERp5	Q15084	2p25.1	4ef0, 3vww, 4gwr, 3w8j, 1x5d
PDILT	PDILT	PDIA7	Q8N807	16p12.3	5xf7, 4nwy
ERP27	ERP27	FLJ32115, PDIA8	Q96DN0	12p12.3	4f9z, 2l4c
ERP29	ERP29	ERp28, ERp31,PDI-DB, PDIA9	P30040	12q24.13	none
ERP44	ERP44	KIAA0573, PDIA10	Q9BS26	9q31.1	none
TMX1	TMX1	TMX, PDIA11	Q9H3N1	14q22.1	1x5e
TMX2	TMX2	PDIA12	Q9Y320	11q12.1	2dj0
TMX3	TMX3	FLJ20793, KIAA1830, PDIA13	Q96JJ7	18q22.1	none
TMX4	TMX4	DJ971N18.2, KIAA1162, PDIA14	Q9H1E5	20p12.3	none
TXNDC5	TXNDC5	MGC3178, FLJ21353, FLJ90810, EndoPDI, Hcc-2, ERp46, PDIA15	Q8NBS9	6p24.3	3wgx, 3wge, 3uvt, 3wgd, 3uj1, 2diz
TXNDC12	TXNDC12	TLP19, ERP18, ERP19, hAG-1, AGR1, PDIA16	O95881	1p32.3	1sen, 2k8v
AGR2	AGR2	XAG-2, HAG-2, AG2, PDIA17	O95994	7p21.1	2lns, 2lnt
AGR3	AGR3	HAG3, hAG-3, BCMP11, PDIA18	Q8TD06	7p21.1	3ph9
DNAJC10	DNAJC10	ERdj5, PDIA19	Q8IXB1	2q32.1	none
CASQ1	CASQ1	PDIB1	P31415	1q23.2	5crg, 5crd, 5crh, 3uom, 5cre
CASQ2	CASQ2	PDIB2	O14958	1p13.1	6owv, 6oww, 2vaf

**Fig. 2:**
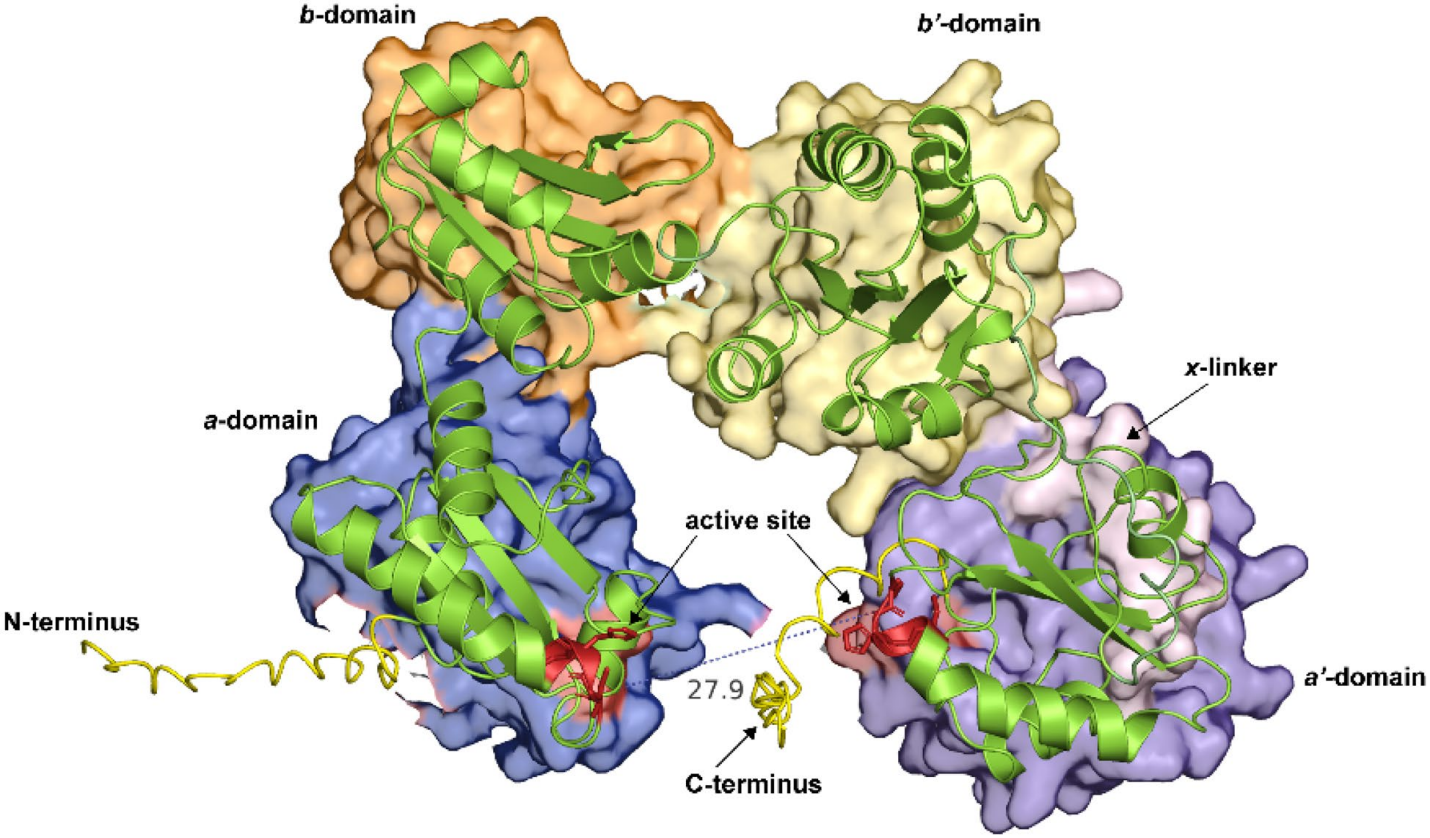
The 3D structure of full-length human PDI/PDIA1 as predicted by AlphaFold (ID:AF-P07237-F1). The major domains are represented by different color surfaces. The active site containing the TRX motif, CGHC is colored in red

It is worth evaluating the structure–function relationships of the prototypic PDI, also referred to as PDIA1 (referred to as PDIA1 hereinafter), which is a 57-kDa dithiol isomerase redox-dependent protein that catalyzes the formation, breakage and rearrangement of the S–S bonds [15]. PDIA1 constitutes  ~ 80% of total ER proteins and was also the first PDI to be identified as a catalyst for protein folding [16, 17]. Structurally, PDIA1 harbors four distinct domains i.e., a, a′, b, b′ [16, 18, 19] (Fig. 2). Other notable structural determinant includes the acidic C-terminal and x-linker (connecting a′ and b′-domains). The TRX-like domain is typically categorized as harboring the a-type (catalytic) and b-type (non-catalytic) domains, whereby the a′ and b′ symbols are often used to indicate the positions of such domains in the polypeptide. The a-type domain contains a thiol group responsible for mediating S–S bond formation, while the b-type domains act as spacers for protein recruitment [20]. The x-linker is a 19-amino acid residue located in between the a′ and b′ domains that are deemed to cushion the substrate-binding on the b′ domain [21, 22].

Other members of the PDI family differ substantially in terms of the a/b-domains configurations and other important motifs (Fig. 1A). Phylogenetic analysis that is based on sequence similarities of PDI reveals the existence of multiple subfamilies, markedly the AGR (TXNDC12, AGR2, AGR3), TMX (TMX1-4) and CASQ (CASQ1, CASQ2) subfamilies (Fig. 1B). The AGR subfamily and some thioredoxin-related transmembrane proteins such as the TMX protein subfamilies only possess the catalytic a-type domain, but not the b-type domain [22, 23]. Another notable feature of PDI proteins is the presence of an ER-retention motif at the uppermost C-terminus end. The founding PDIA1 protein contains a highly acidic tetrapeptide ER-retention motif KDEL (Lys-Asp-Glu-Leu) and is considered a canonical motif for ER retention. This motif is recognized by KDEL receptors in a post-ER compartment and such binding triggers retrieval back to the ER, thus ensuring that the proteins harboring this motif stay in the ER. However, there are variations of this motif (e.g., KTEL, EDEL, QSEL) within the PDI family suggesting that some PDIs have different tolerances in binding affinity to KDEL receptors and thus may escape the ER or are secreted into extracellular space [24]. The CASQ1, CASQ2 ERP27, and ERP29 proteins do not carry the TRX motif and contain only the b-type domains suggesting that they are incapable of mediating S–S bonds. Interestingly, the CASQ subfamily proteins are the only PDI proteins missing the ER-retention motif. Another interesting subset of the PDI family is the more recently discovered transmembrane domain-containing TMX subfamily of proteins (TMX1-4) suggests possible protein localization in the ER membrane or other membranous organelle. Additionally, phylogenetic studies revealed that members of PDI and Rho guanine-dissociation inhibitors (Rho-GDI) have a syntenic linkage and were co-regulated by the same cis-regulatory elements, suggesting that PDI/RhoGDI pairs have a common ancestor and functional linkages [25].

## Core functions of PDI members

The core function of PDI-like protein members is to facilitate the formation of disulfide bonds for secretory and membrane proteins entering the SP, particularly in the ER [26–29]. This ensures that the client proteins are in a proper stabilization and maturation state prior to ER exit. The S–S bond formation occurs primarily in the ER under an oxidative environment, in which they are formed through oxidizing thiol-reactive cysteine and then isomerizing them to attain the right conformation. To understand the formation of disulfide bonds of proteins travelling along with the SP, it is worth reviewing the enzymatic activity of the founding member PDIA1. In the process of native protein folding, PDIA1 undergoes a redox reaction. The oxireduction cycle of PDIA1 alters the conformation to enable correct protein folding. When PDIA1 is in an oxidized state, PDIA1 mediates the enzymatic catalysis of dithiols to disulfides by pairing cysteines in the active site with reduced nascent polypeptides or substrates to form a disulfide bond [30, 31]. The reduced cysteine thiols in the substrate bind to the CGHC disulfide to produce a PDI-protein complex. The complex interacts with a second reduced thiol from the substrate, resulting in an oxidized and stable native protein. Simultaneously, the disulfide in the active site of PDI is reduced to the dithiol state [20]. While PDIA1 is in a reduced state, it catalyzes the breakage of disulfide bonds of the oxidized protein substrates and reduced it to dithiol. To complete the catalytic cycle of PDIA1, the PDIA1 protein must be reoxidized by electron transfer, for example by an electron transport machinery involving an enzyme ER oxidoreductin 1 (Ero1) [32]. Isomerization, on the other hand, occurs during PDIA1 being in a reduced state. The cysteine closest to the N-terminus active site initiates the intramolecular rearrangement of S–S bonds [33]. Client proteins enriched in cysteines are predisposed to error, especially with regard to intramolecular disulfide bonds, hence isomerization is required to convert incorrectly formed disulfides to their native conformation. Isomerization can be thought of a series of reduction and oxidation cycles [15].

PDIs have also been proposed to act as molecular chaperones [34]. A fundamental characteristic of a molecular chaperone is the ability to bind to a partially or completely disordered client protein. When chaperones bind to the substrates, the protein substrates are not in their native functional conformation. Non-native states exist in all proteins in the cell at some point during their life cycle. For example, unfolded polypeptides that have only been translated may bind to chaperones before reaching their native conformation. Chaperones are crucial in maintaining proteostasis. This ability of a molecular chaperone to interact with the misfolded protein is primarily to protect their client protein from aggregation and is often referred to as ‘holdase’ activity. Whereas a process of a chaperone assisting a protein in folding back to its native state is called ‘foldase’ activity. Interestingly, PDIs are shown to be capable of distinguishing between native, unfolded or misfolded proteins through hydrophobic interactions [35]. Misfolded proteins that are lacking disulfide bonds can also bind to PDIs to prevent the aggregation of damaged proteins, suggesting their roles in the ER goes beyond their role in disulfide bond formation [36]. For PDI1A, it has been proposed that the molecular chaperone activities are independent of its redox activity [37, 38]. The C-terminus and a′-domain were shown to be crucial for its chaperone activity. Truncation of the C-terminus and a′ domain results in the loss of the chaperone function, however, it is not known whether this applies to all PDI family members [39, 40].

Since there are multiple PDI family members, their functions may be overlapped. It has been shown that PDI family members may have at least one function as described here, whether it be as oxidases, reductases, isomerases, or molecular chaperones. The differences in structural determinants especially concerning their catalytic thioredoxin fold, hydrophobic pockets, and ER retention motif result in their functions being subspecialized depending on cellular settings. PDI proteins are therefore crucial in ER proteostasis, which aids in the maintenance of several important cellular functions like gluconeogenesis, calcium storage, organelle biogenesis, and lipogenesis. As a result of its various influences on cellular stress, disruption of ER proteostasis frequently leads to the development of multiple disease states.

## Expression of PDI members in cancer

**Fig. 3:**
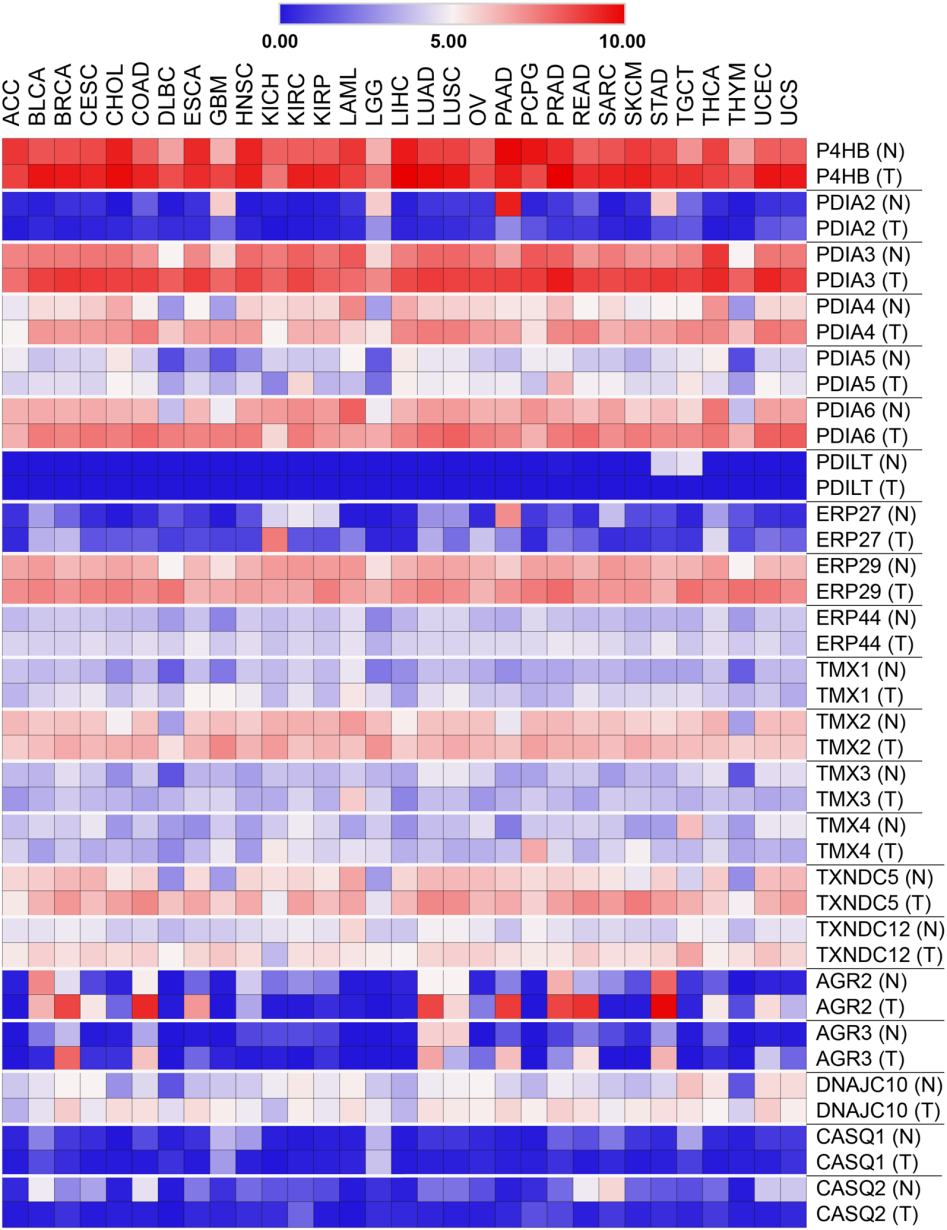
Expression of PDI members in cancer landscape. The expression of PDIs is based on RNA-seq expression data extracted from the TCGA Pan Cancer Atlas

Several PDIs are frequently overexpressed in cancers despite being one of the most abundant cellular proteins in the ER [41]. Assessment of PDI proteins expression using published microarray datasets revealed that PDIA1 is significantly upregulated in the brain and CNS cancers, lymphoma, kidney, ovarian, lung and male germ tumors. Analysis of cytosolic and cell surface proteomes derived from various cancers also yielded similar results [42]. Likewise, other PDI members namely PDIA3, PDIA4, and PDIA6 are also highly expressed in numerous cancer types including breast, thyroid, rectal, gastric and liver cancers [43–45]. Additionally, we analyzed publicly available RNA-Seq data of all PDI members that could give the overall picture of their expression in the cancer landscape. Integration and comparative analysis of TCGA (cancer tissues) and GTEx (normal tissues) RNAseq data showed almost similar trends to previous data in that PDIA1 showed a high level of expression in both normal and cancer tissues suggesting that PDIA1 is a universal catalyst of disulfide bond formation (Fig. 3). The expression of PDIA3, PDIA4, PDIA6, ERP29, and TXNDC5 have also upregulated in most cancer types. On the other hand, PDILT, CASQ1, and CASQ2 proteins are expressed at low levels in most tissues and their expression seems not to be differentially expressed in normal and cancer tissues. Perhaps, the most differentially expressed protein is AGR2 which is upregulated in lung, pancreas, stomach, breast, prostate, and colorectal cancer tissues compared to their normal tissues. It is important to note that these analyses are based on mRNA expression and that proteins can undergo various post-translational modifications (PTMs) that could alter their expression. Current database and proteomic technologies do allow us to comprehensively scrutinize the expression of each PDI. There are several proteomics studies, however, that identified PDI proteins as one of the upregulated proteins in cancer settings. For example, the mass spectrometry-based analysis identified an increased level of PDIA1 in prostate [46], breast [47], and brain [48] tumors. The upregulation of PDIA3 protein expression in colorectal, liver, brain and clear cell ovarian cancer was also revealed by high-throughput OMICS platforms [49–52]. The AGR2 protein was found to be upregulated in esophageal adenocarcinoma as shown by quantitative shotgun proteomics and immunohistochemistry [53].

## Post-translational modifications of PDIs

It has been well-established that the structure, expression, and function of a protein can be altered via PTMs. Therefore, we discuss briefly the PTMs among PDI members that could expand our understanding of their roles. In a recent review, it has been shown that S-nitrosylation, carbonylation and S-glutathionylation can impair the functions of PDIs [54]. These PTMs are aberrant and irreversible, and they are the outcome of cellular nitrosative or oxidative stress, where high levels of reactive nitrogen species (RNS), hydrogen peroxide and reactive oxygen species (ROS) accumulate as modifying agents. Among these PTMs, S-nitrosylation of PDI has been demonstrated to inhibit the chaperone and isomerase activity of PDIs, which have been observed in neurodegenerative diseases [55, 56]. Whereas, S-glutathionylation, the formation of a disulfide bond between glutathione and a cysteine residue of another protein has been attributed to protein misfolding and enhancement of the UPR and has been associated with cancer [57, 58].

Our investigation of the PhosphoSitePlus repository (www.phosphosite.org), on the other hand, revealed that all but one member (ERp27) of the PDI family can undergo extensive native PTMs, encompassing phosphorylation (p), acetylation (Ac), ubiquitylation (Ub) and glycosylation (G), methylation (M) and succinylation (Sc) [59]. To elaborate further, we found that human PDIA1 can accommodate up to 65 PTM sites i.e., Ac (12), G (1), M (7), p (19), Sc (9) and Ub (17). Similar to the other 19 PDI family members, most of these PTMs were identified with large-scale OMICS approaches, notably by mass spectrometry analysis. Of these 65 sites, only 3 sites have been investigated for their biological functions i.e., Thr331p, Ser357p and Ser427p by Yu et al., where they discovered that the phosphorylation of Ser331 and Ser427 affected the conformation and activity of PDIA1 minimally [60]. In contrast, when Ser357 becomes phosphorylated by the secretory pathway kinase Fam20C, PDIA1 adopted an open conformation, which converts PDIA1 from a “foldase” into a “holdase” for preventing protein misfolding in the ER. Besides, the phosphorylation of Ser357 allows PDIA1 to interact with the lumenal domain of IRE1α, a UPR signal transducer to attenuate excessive IRE1α activity. Likewise, 83 PTMs have been reported for PD1A3 in PhosphoSitePlus, including Ac (13), G (4), M (8), p (36), Sc (7) and Ub (15). While it was documented earlier that Lyn, a protein tyrosine kinase of the Src family is primarily responsible for the phosphorylation of tyrosine residues Tyr445, Tyr454 and Tyr467 in PDIA3, little is known about the biological implications of these modifications [61]. PDIA3 is well-known as an essential component for MHC Class I peptide loading complex which presents antigen to CD8 + cytotoxic T cells to enable tumor antigen recognition and anti-tumor immune responses, hence it is vital for cancer immunotherapy [62]. We, therefore, articulate that further functional characterization of the various PTMs located in the PDI family is of critical importance.

## Functions and mechanisms of PDI members in cancer

The high level of expression of PDIs in cancer has been shown to be associated with cancer progression, metastasis and invasion [63, 64]. The mechanisms by which PDIs promote tumour growth and metastasis has been linked with their ability to suppress apoptosis [48, 65, 66]. For example, ERp29 which lacks the catalytic a-type domain has been shown to downregulate eLF2α which in turn upregulates Hsp27, resulting in the inhibition of apoptosis in breast cancer cells. Besides, ERp29 may also promote cancer invasion and metastasis by regulating epithelial-mesenchymal transition (EMT) [63]. Inhibition of PDI activity by the PDI inhibitor, bacitracin increased apoptosis in cancer cells and more evidently in response to ER stress [67]. Similarly, inhibition of PDIA1 activity by PDI inhibitor PACMA 31 reduced the growth of the mouse xenograft model of human OVCAR-8 ovarian cancer [68]. In line with this, siRNA-mediated knockdown of PDI demonstrated caspase-dependent apoptosis in the MCF-7 breast cancer cell line [69]. Meanwhile, overexpression of cytosolic PDIA1 has been shown to inhibit cell death after an apoptotic stimulus [70]. It was also shown that PDIA1 has non-canonical function in supporting ferroptosis in breast cancer cells and induced accumulation of lipid ROS [71]. In lung cancer cells, PDIA4 and PDIA6 were upregulated in response to cisplatin treatment and the inactivation of both proteins directly stimulates cisplatin‐induced apoptosis [72]. This further suggests that these PDIs have a pro-survival role through the regulation of the cell death pathway.

Pin-pointing the exact mechanism by which PDIs contribute to tumorigenesis is impossible because PDIs interact with a wide range of proteins due to their versatile functions and have been observed to contribute to cancer progression via different pathways. The most commonly studied PDI-related signalling pathway is the Ras/Raf/MEK/ERK pathway, which is one of the key signalling pathways involved in the regulation of cell proliferation, survival and differentiation. TXNDC5 is an endo-PDI that can activate the Ras/Raf/MEK/ERK pathway and is required for TNFα-induced angiogenesis [73]. Recently, a study revealed that TXNDC5 high expression was maintained by the SREK1 variant through the interaction of nonsense-mediated decay components in hepatocellular carcinoma [74]. In addition, overexpression of ERp29 may regulate ERK signalling in mesenchymal-like breast cancer cells, which in turn increases E-cadherin and further promotes cellular transition into epithelial properties [75]. PDIA6 was reported to be involved in the Wnt/β-catenin signalling pathway. Overexpression of PDIA6 has been demonstrated to promote the proliferation and cell cycle progression of HeLa cells through activation of Wnt/β-catenin signalling by inhibiting the phosphorylation of β-catenin leading and its proteasomal degradation [76]. Studies using *Caenorhabditis elegans* have linked PDI-associated genes to the Wnt signalling pathway. The PDI-1 ortholog is crucial in specifying neuronal development and that it controls Wnt3a secretion. Mutation of the cysteine in the TRX motif to serine impairs Wnt3a secretion, suggesting that the oxidation and rearrangement of disulfide bonds mediated by PDI are essential during Wnt biogenesis in *C. elegans* [77].

Though normally residing in the ER, PDIs may translocate to the cell surface and catalyze thiol-disulfide exchange in extracellular proteins to assist in the isomerization of disulfide bonds of their interacting proteins such as metalloproteases, selectins and integrins [78]. A previous study has shown that the activation of ADAM17, a disintegrin and metalloprotease which is important for signaling at the cancer cell surface, is modulated by cell surface thiol-isomerases activity of PDIA1 [79]. In addition, PDIA1 can interact with cell surface thyroid hormone receptors via its reductase/isomerase activity suggesting its possible role in thyroid cancers [80]. PDIA1 can also be secreted extracellularly and is capable of mediating cell adhesion and migration, aiding in cancer progression and metastasis by catalyzing thiol-disulfide exchange, which further activates membrane proteins (e.g., integrins) or proteolytic enzymes (e.g., MMPs) [81]. Another cell surface localized PDI member is PDIA6 that is highly expressed at the cell surface of human platelets in response to platelet agonists and can physically interact with the integrin B3 subunit during platelet stimulation [82]. Moreover, PDIA4 protein has been found to be secreted in the serum of Chinese adults with metabolic syndrome which elicit its potential extracellular role in cancer [83]. These findings set new paradigms in that PDI can have gain-of-function activities outside the ER that contribute to the hallmarks of cancer.

Among human PDIs, AGR proteins (TXNDC12, AGR2, and AGR3) are notably associated with oncogenesis. To date, the AGR gene family is the smallest PDI member which has less than 200 amino acids (Fig. 3). Since there are growing interest and studies on AGR proteins (mostly AGR2 and AGR3) in cancer; and their role in the ER and outside the ER, we ought to emphasize our review of this PDI subfamily.

### AGR2

AGR2 is perhaps the most studied PDI family member and is closely related to tumorigenesis. The human homologue named AGR2 was first identified in the EsR-positive breast cancer cell lines [84]. Subsequent OMICS screening identified AGR2 as being overexpressed in the human cancers of the prostate, lung, stomach, ovarian, pancreas, esophagus, as well as head and neck [85]. It has been suggested that AGR2 plays dual roles in cancer development. The first role of ER-based AGR2 is to buffer ER proteostasis in high metabolic and proliferating cancer cells as well as to control protein secretion as widely reviewed previously [85–87]. Elevated AGR2 expression across several cancers has been shown to support the growth of cancer cells through multiple pathways such as Wnt/β-catenin and Hippo signaling pathway [88, 89]. Although AGR2 contains only a single cysteine in its TRX motif, it has been shown that it is capable of forming mixed-disulfide bonds with its substrates [90–92]. AGR2 has also been found outside the ER which was found at the cell surface and secreted into extracellular space. It has been elucidated that extracellularly localized AGR2 contributed to several hallmarks of cancer such as ECM remodeling, inflammation, metastasis, cell proliferation and angiogenesis [93–95]. For example, a recent study demonstrated that the equilibrium of AGR2 monomer–dimer influenced AGR2 secretion and that the secretion yielded pro-inflammatory phenotypes [95]. It has also been found that PDI proteins including AGR2 refluxed to the cytosol in cancer and gain new functions through a phenomenon called ER-to-cytosol Signaling (ERCYS) [96]. The reflux of AGR2 from ER to cytosol results in non-genetic inactivation of the tumor suppressor p53. There were also multiple interactomics studies aimed at expanding the search for AGR2 interaction partners to further understand the role of AGR2 in cancer. From these studies, AGR2 seems to interact largely with secreted and cell-surface proteins such as EpCAM, MUC5AC, EGFR and PROD1, suggesting that AGR2 is predominantly involved in their maturation and that the binding is crucial for their signaling [97–100].

### TXNDC12 (AGR1)

TXNDC12, also known as AGR1 was initially identified from the ER lumen of healthy mice using a proteomics screen and was initially named ERp19 [101] (Table 1). TXNDC12 is the only protein within the AGR subfamily to contain dual cysteines, CGHC in the catalytic TRX motif. Interestingly, a phylogenetic tree of AGR genes across different classes of vertebrates showed that TXNDC12 is present exclusively in lower vertebrates, but lost in higher evolutionary trees [102]. TXNDC12 is believed to be the founding gene of the AGR family. Structural and biophysical studies have revealed that TXNDC12 has oxidase and isomerase activity [103–105]. TXNDC12 has been reported to form mixed disulfide bonds with substrates in the ER and in this case, TXNDC12 shows specificity to pentraxin-related protein, PTX3 during its assembly into a decamer [106]. Ectopic expression of TXNDC12 can be detected in both cell lysates and culture medium from cell lines, suggesting that this protein can be secreted extracellularly, however, the extracellular function of TXNDC12 has not been documented. TXNDC12 has also not been identified in OMICS screens of human cancer until recently. Immunohistochemical analysis and qRT-PCR of gastric cancer tissues and cell lines showed that TXNDC12 is upregulated in tumors compared to normal tissues [107]. Patients with heavy staining of TXNDC12 demonstrate poor prognoses and a positive correlation with both tumor size and lymph node metastases. TXNDC12 can also promote proliferation, migration, and invasion of gastric cancer cells while TXNDC12 knockdown significantly abolishes these effects. Interestingly overexpression of TXNDC12 significantly enhanced FAK phosphorylation at Tyr-397 and paxillin phosphorylation at Tyr118, suggesting the role of TXNDC12 in the FAK/paxillin pathway in gastric cancer [107]. This study was the first study to show that TXNDC12, like other AGR homologues, is involved in tumorigenesis at least in the gastric cancer population while its role in other types of cancer warrants further investigation.

### AGR3

AGR3 is the smallest member of the PDI family containing only 166 amino acids. It was discovered as a new protein whose sequence is highly homologous to AGR2 in a proteomics analysis from enriched plasma membrane proteins of breast cancer cell lines and was initially named BCMP11 [108]. Fluorescently labelled AGR3 demonstrated that the protein localized in secretory or endosome-like organelles in the breast cancer cell line [108]. This is another evidence of PDI member that can be captured in the plasma membrane of cancerous cell; the localization resembles a secretory molecule thus suggesting that it may function as a ligand for plasma membrane-associated proteins or maybe secreted for extracellular function. Furthermore, the same research group showed that AGR2 and AGR3 were both co-expressed in estrogen receptor (EsR)-positive breast cancer and can physically interact with LY6/PLAUR Domain Containing 3 (LYPD3 /C4.4a) and extracellular alpha-dystroglycan (DAG-1) [109]. It is also interesting to note that both the AGR2 and AGR3 genes are located side-by-side in chromosome 7p21 and are in close proximity to EsR binding sites, therefore it is not surprising that the genes are co-expressed particularly in EsR positive breast cancer [110]. AGR3 was found to be expressed cytosolically in four human ovarian cancer subtypes; serous papillary, endometrioid, clear cell, and mucinous where the latter showed the highest AGR3 expression [111]. The expression of AGR3 was found to be associated with EsR-negative ovarian cancer. In different studies, the AGR3 protein was found to be overexpressed in serous ovarian cancer and high expression of AGR3 in the serous subtype is correlated with poor prognosis [50, 112]. This result contradicts the previous study which reported that AGR3 expression is confined to the mucinous type [111]. AGR3 can also be secreted outside the cell as it was found to be secreted into blood serum and can be used as an independent prognostic factor for early screening of breast cancer patients [113]. AGR2 and AGR3 expression are coupled in EsR positive breast cancer and the combination of AGR2 and AGR3 as biomarkers resulted in increased sensitivity and specificity thus suggesting that they can be used as a prognostic factor [113]. The crystal structure of AGR3 (PDB code:3PH9) has revealed that it can form an asymmetric unit consisting of two AGR3 molecules, however, there was no evidence that AGR3 can form a spontaneous dimer in solution [114].

## PDI regulation during ER stress

The ER governs a unique environment to establish a balanced ER proteostasis. This ER proteostasis can be perturbed by physiological as well as multiple environmental and cellular signals such as high protein demand, viral infections, environmental toxins, inflammatory cytokines, and mutant protein expression resulting in an accumulation of misfolded and unfolded proteins in the ER lumen, a condition termed as ER stress [115]. It is known that under ER stress, cells activate a series of complementary adaptive mechanisms to cope with increased demands of protein folding in the ER. This adaptive system is called the unfolded protein response (UPR) which is a highly conserved signal transduction pathway to reduce unfolded/misfolded protein load and restore proteostasis [116]. When unfolded proteins accumulate in the ER, ER-resident proteins including PDIs become occupied, releasing transmembrane ER protein sensors involved in inducing the UPR. There are three key mammalian UPR sensors that comprise the endoribonuclease inositol-requiring enzyme 1-alpha (IRE1α), protein kinase RNA-like endoplasmic reticulum kinase (PERK) and activating transcription factor 6 (ATF6α) [115]. During physiological stress, upon sensing an imbalance in compartmental proteostasis, these sensors will transmit stress signals from the ER lumen to downstream signalling. This helps to reduce newly-synthesized proteins, thus facilitating the efficient degradation of superfluous and misfolded proteins in the ER [117].

An important link between ER and tumour development has been established recently [118–121]. In transformed cells like cancer, the SP is exposed to a strong environmental pressure such as hypoxia, oxidative stress or chemotherapies, aneuploidy, and high proliferation rates which together contribute to tumorigenesis. There also seems to be crosstalk between UPR with ER-associated degradation (ERAD) and autophagy that work together to remove unfolded/misfolded proteins or protein aggregates from the ER [116, 122]. The regulation by UPR is usually short-term, and prolonged activation of UPR might otherwise induce apoptosis. The prolonged activation of UPR that leads to ER stress-induced apoptosis is activated via three primary pathways, including the IRE1/ASK1/JNK pathway, caspase-12 kinase pathway, and the C/EBP homologous protein (CHOP)/GADD153 pathway [123, 124].

The upregulation of PDIs is generally accompanied by the overexpression of UPR-related proteins such as BiP, GRP, IRE1α, ATF6 α and elF2a [125, 126]. For example, AGR2 expression can be controlled by the UPR upon ER stress and most likely depends on both IRE1α and ATF6α signalling [127]. Knockdowns of IRE1α and ATF6α resulted in decreased basal AGR2 mRNA expression and also prevented AGR2 induction upon chemical ER stressor treatment. Under ER stress, PDIA5 is crucial for the formation of S–S bonds in ATF6α leading to its export from the ER and activation of its target genes [128]. PDIA6 has been shown to directly interact with IRE1α and PERK and inactivates them, resulting in the limitation of UPR signalling within the physiological range [129–131].

## Targeting PDIs for cancer therapeutics

It is evident that the high expression of some members of the PDI family supports cancer growth and thus gives them diagnostic/prognostic utility. This also suggests that targeting them is relevant to abrogate cancer. Interestingly, some PDIs can be secreted into the bodily fluids of cancer patients such as serum, plasma, and urine which fits a criterion to be a strong biomarker candidate. Clinical detection of PDI levels in serum could reflect PDI expression in cancer patients, thus pointing to a potential diagnostic/prognostic significance. PDIA1 has been used as a serum marker for the diagnosis of colorectal cancer [132]. Moreover, in patients with melanoma or refractory acute myeloid leukemia, PDIA1 is an immunogenic molecule that is targeted by antibodies during immune-mediated tumor breakdown [133, 134]. The same study also showed that PDIA6 can also elicit a similar antibody response, suggesting the therapeutic potential of PDIA1 and PDIA6 in hematologic malignancies. Moreover, a recent study showed that PDI inhibition resulted in the suppression of tumor cell growth and improve T cell tumor control [135].

It is worth noting that some PDIs are expressed at high levels even in normal cells (e.g., PDIA1) to maintain housekeeping activities such as substrate folding and to react to UPR signaling. Therefore, targeting these PDIs may need pharmacokinetics fine-tuning to prevent adverse systemic effects. Alternatively, a drug delivery system could be developed to specifically target PDI in cancer cells. Another important aspect to consider is that the expression landscape of PDI is different in cancer, thus targeting PDI may only work in specific types of cancer. This has been shown in the case of PDI inhibitor bacitracin which works well in inducing apoptosis in skin, breast and brain cancer cells but not in cervical cancer cells [67, 69]. For clinical applications, the most common treatment for cancer is chemotherapy. Unfortunately, chemotherapy resistance is one of the challenges in clinical cancer treatment, as several studies have shown that PDI plays a role in mediating chemoresistance in several types of cancers. For instance, it is reported that PDIA1 protein is overexpressed in ovarian cancer HeLa cells in response to aplidin, contributing to resistance to this drug. The drug resistance in the cells was abrogated through PDIA1 inhibitor bacitracin thus, implicating PDIA1 as a contributing factor to aplidin resistance. Fortunately, these data imply that combining PDI inhibitors with traditional anti-cancer compounds could resolve the chemoresistance issue and might even attain synergetic effects [136].

We summarized several other PDI agents that have been experimented on previously, their mode of action and their effectiveness in treating cancer cells in Table 2. These include antibiotics [137], sulfhydryl blockers [138, 139], arsenical [140], natural and synthetic estrogenic compounds [141]. Additionally, we searched the Drugbank database (https://go.drugbank.com/) for potential drugs that could potentially be effective or repurposed to target all 21 members of the PDI family (Table 3). Only three PDI members namely, PDIA1, CASQ1, and PDIA3 could be targeted by drugs in this repository. Ribostamycin is a WHO-approved broad-spectrum antibiotic for the human application that has been repurposed to potentially inhibit PDIA1. The antibiotic was shown to inhibit PDIA1 chaperone activity but not its isomerase activity; however, the pharmacological action of the drug has yet to be elucidated [142].

**Table 2:** Small molecule PDI inhibitors

Name		IC_50_ (micromolar)	Selectivity	Reversibility	PDI inhibition activity		References
					In-vitro	In-vivo	
*Acrolein*	10 (in pH 6.3)		Nonspecific	Irreversible	N/A	N/A	[143]
*Bacitracin*	90		Nonspecific	Irreversible	•Apoptosis increased together with other drugs in melanoma cells	The neuroprotective effect of 4-HBA was nullified	[144–148]
*Bacitracin A *(major analogue)	590				•Glioma cell progression and invasion were reduced		[149, 150]
*Bacitracin B*	1050				•Inhibits virus entry		[149–153]
*Bacitracin F*	20				•Inhibits platelet accumulation		[154, 155]
*Bacitracin H*	40				•Aggregation of Cu/Zn superoxide dismutase increases •Inhibits VKORC1 activity •Transcriptional activity of NF-kB increases •Inhibits NADPH oxidase activity •Inhibits diphtheria toxin cytotoxicity •Inhibits thyroid-stimulating hormone receptor shedding		[156–161]
*Bepristat 2a*	0.3–2.1		b′ domain of PDIA1	Reversible	•Prevent adhesion of MDA-MB-231 and MCF-7 cells to collagen, endothelium and fibronectin •Reduce cancer cell trans-endothelial migration	Impair platelet aggregation	[162, 163]
*Cysteamine*	66		Nonspecific	Irreversible	•Prevents polyQ-induced apoptosis in a PC12 cell model of Huntington’s Disease		[164]
*DNTB*	100		Nonspecific	Irreversible	•Inhibits diphtheria toxin activation •Prevents H9 cells from HIV-1 infection (IC_50_ = 0.3 mM) •Long-term host cell protection at the late stages of the viral cycle		[153, 160, 165]
*Estrogens: E*_*1*_, *E*_*2*_*, DES* and *E*_*3*_	At 1 micromolar > 30% inhibition		Nonspecific	Reversible			[166]
*Iodoacetamide*	8 (in pH 6)		Nonspecific	Irreversible			[143]
*Juniferdin*	0.156–3		PDIA3 and PDIA4	Reversible	•Inhibits PDI-catalyzed reduction of HIV gp120 and viral entry •Cytotoxic in HeLa, HepG2, HT1080 and K562 cells		[167]
*NEM*	8		Nonspecific	Irreversible			[143]
*PACMA 31*	10		Shows selectivity for Cys53 of a′ domain	Irreversible	•Cytotoxic in ovarian cancer cell lines (OVCAR-8, NCI/ADR-RES, HEY and OVCAR-3)	Accumulates in tumour and suppresses tumour growth in a mouse xenograft model of ovarian cancer; no significant toxicity towards normal tissues	[168]
*PAO*	85		Low specificity reaction with CXXC motif	Irreversible	•Induces rapid shedding of L-selectin from isolated neutrophils •Inhibits PDI-catalyzed reductive release of acid-soluble [^125^I] tyramine-SH from surface-bound [^125^I] tyramine-SS-poly(D-lysine) (IC_50_ = 10 micromolar) •Effective before or during HIV-1 infection •Non-effective after infection progressed in P4, PM1, H9, 1G5 and macrophage-depleted peripheral blood monocytic cells		[168–172]
*Quercetin-3-rutinoside *(*rutin*)	6.1		PDIA3, PDIA4, PDIA6, thioredoxin and thioredoxin reductase	Reversible	•Inhibits platelet aggregation and endothelial cell-mediated fibrin generation	Inhibits thrombus formation	[146, 173]
*Quercetin-3-glucoside *(*isoquercetin*)	9.2		Plasma PDI	Reversible		•Increased plasma PDI inhibitory activity •Decreases thrombin production	[174, 175]
*RB-11-ca*	40			Irreversible	•Cytotoxic in HeLa cells with EC_50_ = 23.9 micromolar		[176]
*Ribostamycin*	Sufficient inhibition of PDI with a molar ratio of 100:1 (Kd = 3.19 × 10^–4^ M)		Nonspecific	Reversible			[142]
*Thiomuscimol*	23		Nonspecific	Irreversible	•Prevents polyQ-induced apoptosis in a PC12 cell model of Huntington’s Disease		[164]
*16F16*	63			Irreversible	•Inhibits apoptosis in a PC12 cell model of Huntington's Disease	Prevents neurotoxicity in medium spiny neurons in the striatal region of brain slices	[164]
*E64FC26*	1.9–25.9		PDIA1, PDIA3, PDIA4, TXNDC5, PDIA6		•Inhibit tumor cell growth and T cell tumor control	Improved survival and enhanced the activity of bortezomib in multiple mywloma	[135, 177]
*CCF642-34*	< 5		PDIA1		•Induces apoptosis in myeloma cells		[178, 179]

**Table 3:** Approved and investigational drugs targeting PDIs

Target	Drug name
PDIA1	Ribostamycin, copper, zinc, artenimol, zinc acetate, zinc chloride, zinc sulfate
CASQ1	Calcium citrate, calcium levulinate, calcium phosphate, calcium phosphate dihydrate
PDIA3	Copper, zinc, zinc acetate, zinc chloride, zinc sulfate
CASQ2	Calcium citrate, calcium phosphate, calcium phosphate dihydrate

As discussed previously, AGR2 is the most studied PDI member in the cancer setting and therefore, researchers have a keen interest in targeting this molecule for cancer treatment. For example, the AGR2 mouse monoclonal antibody has already been developed and was shown to suppress the growth of breast cancer cells [180]. A further study developed a humanized version of the same antibody and demonstrated that the antibody was able to inhibit tumor growth in an ovarian cancer xenograft model [181]. Another study had developed the same monoclonal antibody was also able to inhibit lung cancer growth, suppressed tumor metastasis and prolong survival in mice [182]. Additionally, monoclonal antibodies in the form of the synthetic single-chain variable fragment (scFv) isolated from antibody phage display are currently being developed and current data demonstrated that the scFv targeting AGR2 was able to bind the N-terminal of AGR2 with high affinity [183].

## Concluding remarks

The founding member PDIA1 was discovered almost 60 years ago [184], and the idea of the PDI family was introduced in 1994 with only 4 members [185]. Since then, the family has been expanded to include new members with different structural features, sizes and functions but having the capability to assist in protein folding especially vis-à-vis disulfide bond formation. Owing to this capability, artificial mini proteins have been developed to resemble the PDI-like catalytic activity for promoting the folding process in preparations of peptide-based drugs [186]. Though PDI proteins are crucial in the normal physiology of the ER by ensuring proper folding and maturation of substrate proteins before they are exported to their destined locations, it has been found that several members of the PDI play roles in cancer as discussed in the review. It is not surprising that some PDIs are elevated in cancer to cater for the rapid growth of cancer cells due to higher protein synthesis demand in cancer as opposed to normal cells. PDI proteins have also been implicated in a wide range of diseases other than cancer including neurodegenerative, cardiovascular, inflammatory bowel, infectious, metabolic and bone diseases [187–189]. These diseases are caused or associated with protein misfolding, accumulation, aggregation or assembly that requires elevated and robust folding catalysts like PDIs. Therefore, the development of drugs that target these pathways will be of benefit not only for cancer therapy but would be applicable for these other associated diseases as well.

**Fig. 4:**
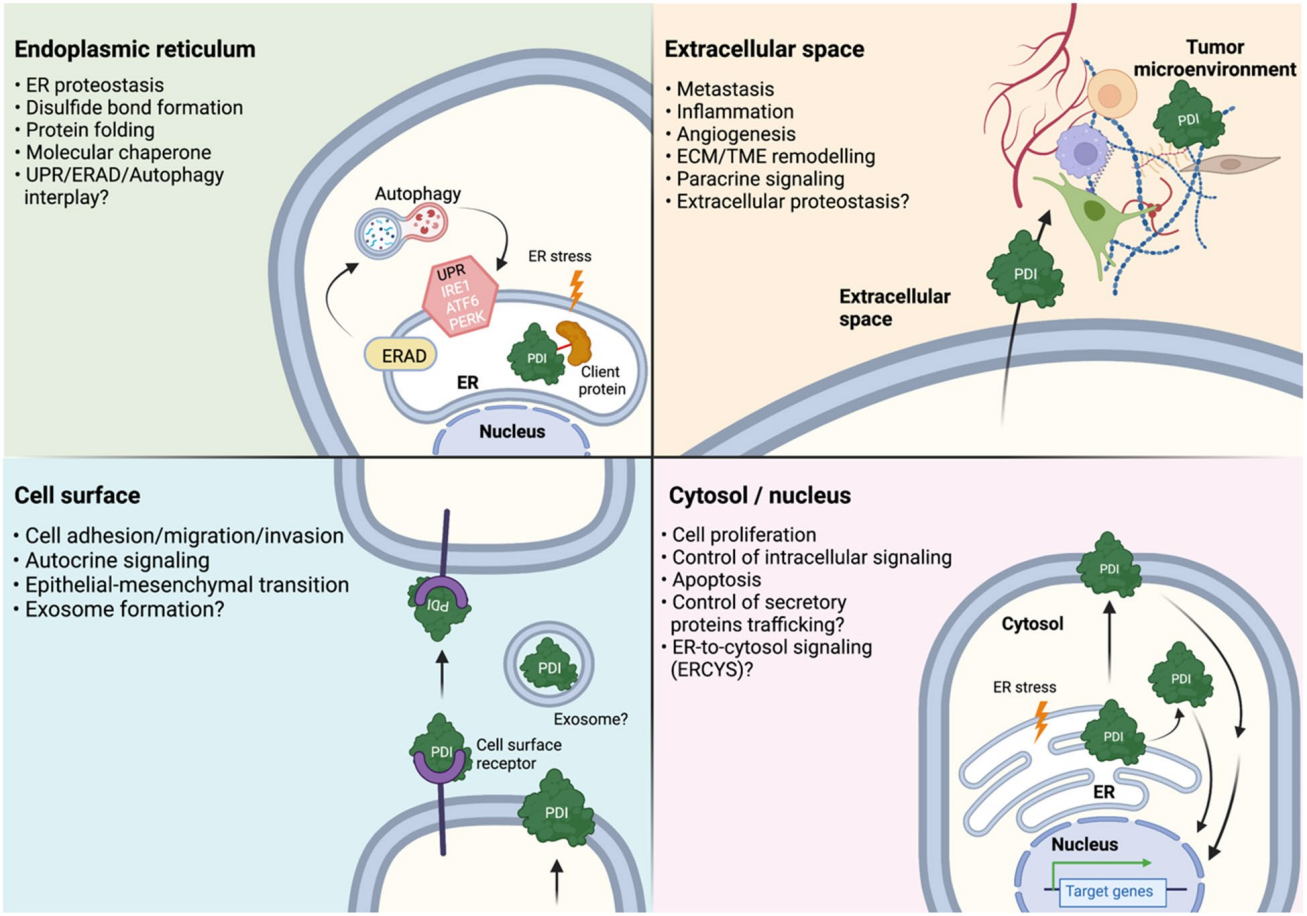
Emerging roles of PDI protein family in cancer. Schematic representation summarizing the existing and emerging roles of PDI proteins in cancer. Although PDI proteins are crucial for ER function and integrity in normal cell physiology, in cancer cells however, PDI proteins can be translocated into different sub-cellular localizations such as the cytosol, cell surface and extracellular milieu. Existing data showed that these non-ER localizations of PDI proteins gain new functions in supporting cancer growth suggesting that the roles of PDI proteins go beyond the ER in diseased state like cancer

The known and proposed roles PDI proteins in cancer setting are summarized in Fig. 4. Attention is now turning to the non-ER localized function and in particular the roles of cell surface and secreted PDI proteins [83, 190, 191]. This is interesting because PDI proteins are crucial for ER function and integrity, and the localization of PDI proteins outside the ER has sparked an interest in the field because it has been shown that the presence of selected PDI proteins in extracellular can contribute to the emergence of cancer. The extracellular roles of PDI need to be further examined to isolate the key players and pathways that can contribute to cancer. Another aspect that can be pursued is to closely look at the three-dimensional (3D) structure of each PDIs. The advancement of high-resolution structural biology techniques like cryogenic electron microscopy and structural bioinformatics using prediction-based methods (e.g., AlphaFold [192]) could give additional insight into PDI enzymatic and chaperone activities. Moreover, cancer cells could be highly dependent on molecules or pathways that support their growth and progression. Therefore, targeting the interfaces between proteins (i.e., protein–protein interaction network) had been proposed as a cancer therapeutic option. In this case, it is worth it to (1) expand the PDIs client proteins and identify the PDIs protein–protein interaction hubs; and (2) screen and validate drugs/inhibitors that could collapse these protein–protein interaction interfaces. Also, gene targeting technologies such as the CRISPR-based genome editing tools could be employed to identify the PDIs’ downstream targets in cancer cells. Finally, significant attention should also be drawn to the other PDI family members, especially to those that are least studied. A comprehensive understanding of the biology of the PDI family members could shed light on how PDIs deregulation would promote the pathogenesis of cancers as well as other related protein-folding diseases.

## Data Availability

Not applicable.

## References

[1] Barlowe CK, Miller EA (2013). Secretory protein biogenesis and traffic in the early secretory pathway. Genetics.

[2] Kumar R, Kumari B, Kumar M (2017). Prediction of endoplasmic reticulum resident proteins using fragmented amino acid composition and support vector machine. PeerJ.

[3] Ellgaard L, Ruddock LW (2005). The human protein disulphide isomerase family: substrate interactions and functional properties. EMBO Rep.

[4] Bošnjak I, Bojović V, Šegvić-Bubić TS, Bielen A (2014). Occurrence of protein disulfide bonds in different domains of life: a comparison of proteins from the Protein Data Bank. Protein Eng Des Sel.

[5] Balch WE, Morimoto RI, Dillin A, Kelly JW (2008). Adapting proteostasis for disease intervention. Science.

[6] Hipp MS, Kasturi P, Hartl FU (2019). The proteostasis network and its decline in ageing. Nat Rev Mol Cell Biol.

[7] Gidalevitz T, Ben-Zvi A, Ho KH, Brignull HR, Morimoto RI (2006). Progressive disruption of cellular protein folding in models of polyglutamine diseases. Science.

[8] Gidalevitz T, Krupinski T, Garcia S, Morimoto RI (2009). Destabilizing protein polymorphisms in the genetic background direct phenotypic expression of mutant SOD1 toxicity. PLoS Genet.

[9] Gidalevitz T, Kikis EA, Morimoto RI (2010). A cellular perspective on conformational disease: the role of genetic background and proteostasis networks. Curr Opin Struct Biol.

[10] Cohen FE, Kelly JW (2003). Therapeutic approaches to protein-misfolding diseases. Nature.

[11] Galligan JJ, Petersen DR (2012). The human protein disulfide isomerase gene family. Hum Genom.

[12] Persson S, Rosenquist M, Knoblach B, Khosravi-Far R, Sommarin M, Michalak M (2005). Diversity of the protein disulfide isomerase family: identification of breast tumor induced Hag2 and Hag3 as novel members of the protein family. Mol Phylogenet Evol.

[13] Laurindo FRM, Pescatore LA, de Castro FD (2012). Protein disulfide isomerase in redox cell signaling and homeostasis. Free Radic Biol Med.

[14] Appenzeller-Herzog C, Ellgaard L (2008). The human PDI family: versatility packed into a single fold. Biochim Biophys Acta Mol Cell Res.

[15] Wilkinson B, Gilbert HF (2004). Protein disulfide isomerase. Biochim Biophys Acta Proteins Proteom.

[16] Ferrari DM, Söling H-D (1999). The protein disulphide-isomerase family: unravelling a string of folds. Biochem J.

[17] Goldberger RF, Epstein CJ, Anfinsen CB (1964). Purification and properties of a microsomal enzyme system catalyzing the reactivation of reduced ribonuclease and lysozyme. J Biol Chem.

[18] Hatahet F, Ruddock LW (2007). Substrate recognition by the protein disulfide isomerases. FEBS J.

[19] Hogg PJ (2003). Disulfide bonds as switches for protein function. Trends Biochem Sci.

[20] Hudson DA, Gannon SA, Thorpe C (2015). Oxidative protein folding: from thiol–disulfide exchange reactions to the redox poise of the endoplasmic reticulum. Free Radic Biol Med.

[21] Nguyen VD, Wallis K, Howard MJ, Haapalainen AM, Salo KEH, Saaranen MJ (2008). Alternative conformations of the x region of human protein disulphide-isomerase modulate exposure of the substrate binding b’ domain. J Mol Biol England.

[22] Wang C, Chen S, Wang X, Wang L, Wallis AK, Freedman RB (2010). Plasticity of human protein disulfide isomerase: evidence for mobility around the X-linker region and its functional significance. J Biol Chem.

[23] Bekendam RH, Bendapudi PK, Lin L, Nag PP, Pu J, Kennedy DR (2016). A substrate-driven allosteric switch that enhances PDI catalytic activity. Nat Commun.

[24] Jordan PA, Gibbins JM (2006). Extracellular disulfide exchange and the regulation of cellular function. Antioxidants Redox Signal.

[25] Moretti AIS, Pavanelli JC, Nolasco P, Leisegang MS, Tanaka LY, Fernandes CG (2017). Conserved gene microsynteny unveils functional interaction between protein disulfide isomerase and rho guanine-dissociation inhibitor families. Sci Rep.

[26] Schwaller M, Wilkinson B, Gilbert HF (2003). Reduction-reoxidation cycles contribute to catalysis of disulfide isomerization by protein-disulfide isomerase. J Biol Chem.

[27] Rajpal G, Arvan P, Kastin AJ (2013). Chapter 236—disulfide bond formation. Handbook of biologically active peptides.

[28] Hatahet F, Ruddock LW (2009). Protein disulfide isomerase: a critical evaluation of its function in disulfide bond formation. Antioxid Redox Signal.

[29] Kobayashi Y, Oguro A, Hirata Y, Imaoka S (2021). The regulation of hypoxia-inducible factor-1 (HIF-1alpha) expression by protein disulfide isomerase (PDI). PLoS ONE.

[30] Walker KW, Gilbert HF (1995). Oxidation of kinetically trapped thiols by protein disulfide isomerase. Biochemistry.

[31] Forrester MT, Benhar M, Stamler JS (2006). Nitrosative stress in the ER: a new role for S-nitrosylation in neurodegenerative diseases. ACS Chem Biol.

[32] Zheng J, Gilbert HF (2001). Discrimination between native and non-native disulfides by protein-disulfide isomerase. J Biol Chem.

[33] Xu S, Sankar S, Neamati N (2014). Protein disulfide isomerase: a promising target for cancer therapy. Drug Discov Today.

[34] McLaughlin SH, Bulleid NJ (1998). Thiol-independent interaction of protein disulphide isomerase with type X collagen during intra-cellular folding and assembly. Biochem J.

[35] Klappa P (1998). The b′ domain provides the principal peptide-binding site of protein disulfide isomerase but all domains contribute to binding of misfolded proteins. EMBO J.

[36] Puig A, Gilbert HF (1994). Protein disulfide isomerase exhibits chaperone and anti-chaperone activity in the oxidative refolding of lysozyme. J Biol Chem.

[37] Freedman RB, Desmond JL, Byrne LJ, Heal JW, Howard MJ, Sanghera N (2017). “Something in the way she moves”: the functional significance of flexibility in the multiple roles of protein disulfide isomerase (PDI). Biochim Biophys Acta Proteins Proteom.

[38] Perri ER, Thomas CJ, Parakh S, Spencer DM, Atkin JD (2016). The unfolded protein response and the role of protein disulfide isomerase in neurodegeneration. Front Cell Dev Biol.

[39] Cao SS, Zimmermann EM, Chuang B, Song B, Nwokoye A, Wilkinson JE (2013). The unfolded protein response and chemical chaperones reduce protein misfolding and colitis in mice. Gastroenterology.

[40] Tian R, Li S-J, Wang D-L, Zhao Z, Liu Y, He R-Q (2004). The acidic C-terminal domain stabilizes the chaperone function of protein disulfide isomerase. J Biol Chem.

[41] Powell LE, Foster PA (2021). Protein disulphide isomerase inhibition as a potential cancer therapeutic strategy. Cancer Med.

[42] Shin BK, Wang H, Yim AM, Le Naour F, Brichory F, Jang JH (2003). Global profiling of the cell surface proteome of cancer cells uncovers an abundance of proteins with chaperone function. J Biol Chem.

[43] Uyy E, Suica VI, Boteanu RM, Manda D, Baciu AE, Badiu C (2016). Endoplasmic reticulum chaperones are potential active factors in thyroid tumorigenesis. J Proteome Res.

[44] Xu X, Wei X, Ling Q, Cheng J, Zhou B, Xie H (2011). Identification of two portal vein tumor thrombosis associated proteins in hepatocellular carcinoma: protein disulfide-isomerase A6 and apolipoprotein A-I. J Gastroenterol Hepatol.

[45] Ramos FS, Serino LTR, Carvalho CMS, Lima RS, Urban CA, Cavalli IJ (2015). PDIA3 and PDIA6 gene expression as an aggressiveness marker in primary ductal breast cancer. Genet Mol Res.

[46] Alaiya AA, Al-Mohanna M, Aslam M, Shinwari Z, Al-Mansouri L, Al-Rodayan M (2011). Proteomics-based signature for human benign prostate hyperplasia and prostate adenocarcinoma. Int J Oncol.

[47] Thongwatchara P, Promwikorn W, Srisomsap C, Chokchaichamnankit D, Boonyaphiphat P, Thongsuksai P (2011). Differential protein expression in primary breast cancer and matched axillary node metastasis. Oncol Rep.

[48] Goplen D, Wang J, Enger PØ, Tysnes BB, Terzis AJA, Laerum OD (2006). Protein disulfide isomerase expression is related to the invasive properties of malignant glioma. Cancer Res.

[49] Takata H, Kudo M, Yamamoto T, Ueda J, Ishino K, Peng W-X (2016). Increased expression of PDIA3 and its association with cancer cell proliferation and poor prognosis in hepatocellular carcinoma. Oncol Lett.

[50] Samanta S, Tamura S, Dubeau L, Mhawech-Fauceglia P, Miyagi Y, Kato H (2017). Expression of protein disulfide isomerase family members correlates with tumor progression and patient survival in ovarian cancer. Oncotarget.

[51] Yang Z, Liu J, Shi Q, Chao Y, Di Y, Sun J (2018). Expression of protein disulfide isomerase A3 precursor in colorectal cancer. OTT Dove Press.

[52] Tu Z, Ouyang Q, Long X, Wu L, Li J, Zhu X (2022). Protein disulfide-isomerase A3 is a robust prognostic biomarker for cancers and predicts the immunotherapy response effectively. Front Immunol.

[53] O’Neill JR, Pak H-S, Pairo-Castineira E, Save V, Paterson-Brown S, Nenutil R (2017). Quantitative shotgun proteomics unveils candidate novel esophageal adenocarcinoma (EAC)-specific proteins. Mol Cell Proteom.

[54] Mutus B, Sexton D, Atkin JD, Parakh S (2015). Novel roles for protein disulphide isomerase in disease states: a double edged sword?. Front Cell Dev Biol.

[55] Nakamura T, Lipton SA (2011). S-nitrosylation of critical protein thiols mediates protein misfolding and mitochondrial dysfunction in neurodegenerative diseases. Antioxid Redox Signal.

[56] Uehara T, Nakamura T, Yao D, Shi Z-Q, Gu Z, Ma Y (2006). S-Nitrosylated protein-disulphide isomerase links protein misfolding to neurodegeneration. Nature.

[57] Townsend DM, Manevich Y, He L, Xiong Y, Bowers RR, Hutchens S (2009). Nitrosative-stress induced S-glutathionylation of PDI leads to activation of the unfolded protein response. Cancer Res.

[58] Neves RPP, Fernandes PA, Ramos MJ (2017). Mechanistic insights on the reduction of glutathione disulfide by protein disulfide isomerase. Proc Natl Acad Sci USA.

[59] Hornbeck PV, Zhang B, Murray B, Kornhauser JM, Latham V, Skrzypek E (2015). PhosphoSitePlus, 2014: mutations, PTMs and recalibrations. Nucleic Acids Res.

[60] Yu J, Li T, Liu Y, Wang X, Zhang J, Wang X (2020). Phosphorylation switches protein disulfide isomerase activity to maintain proteostasis and attenuate ER stress. EMBO J.

[61] Donella-Deana A, James P, Staudenmann W, Cesaro L, Marin O, Brunati AM (1996). Isolation from spleen of a 57-kDa protein substrate of the tyrosine kinase lyn. Eur J Biochem.

[62] Reeves E, James E (2017). Antigen processing and immune regulation in the response to tumours. Immunology.

[63] Zhang D, Tai LK, Wong LL, Chiu L-L, Sethi SK, Koay ESC (2005). Proteomic study reveals that proteins involved in metabolic and detoxification pathways are highly expressed in HER-2/neu-positive breast cancer. Mol Cell Proteom.

[64] Zong J, Guo C, Liu S, Sun M-Z, Tang J (2012). Proteomic research progress in lymphatic metastases of cancers. Clin Transl Oncol.

[65] Yang S, Jackson C, Karapetyan E, Dutta P, Kermah D, Wu Y (2022). Roles of protein disulfide isomerase in breast cancer. Cancers.

[66] Ma Y-S, Feng S, Lin L, Zhang H, Wei G-H, Liu Y-S (2021). Protein disulfide isomerase inhibits endoplasmic reticulum stress response and apoptosis via its oxidoreductase activity in colorectal cancer. Cell Signal.

[67] Lovat PE, Corazzari M, Armstrong JL, Martin S, Pagliarini V, Hill D (2008). Increasing melanoma cell death using inhibitors of protein disulphide isomerases to abrogate survival responses to endoplasmic reticulum stress. Cancer Res.

[68] Xu S, Butkevich AN, Yamada R, Zhou Y, Debnath B, Duncan R (2012). Discovery of an orally active small-molecule irreversible inhibitor of protein disulfide isomerase for ovarian cancer treatment. Proc Natl Acad Sci.

[69] Hashida T, Kotake Y, Ohta S (2011). Protein disulfide isomerase knockdown-induced cell death is cell-line-dependent and involves apoptosis in MCF-7 cells. J Toxicol Sci.

[70] Na KS, Park BC, Jang M, Cho S, Lee DH, Kang S (2007). Protein disulfide isomerase is cleaved by caspase-3 and -7 during apoptosis. Mol Cells.

[71] Wang H, Wang P, Zhu BT (2022). Mechanism of erastin-induced ferroptosis in MDA-MB-231 human breast cancer cells: evidence for a critical role of protein disulfide isomerase. Mol Cell Biol.

[72] Tufo G, Jones AWE, Wang Z, Hamelin J, Tajeddine N, Esposti DD (2014). The protein disulfide isomerases PDIA4 and PDIA6 mediate resistance to cisplatin-induced cell death in lung adenocarcinoma. Cell Death Differ.

[73] de Camargo LL, Babelova A, Mieth A, Weigert A, Mooz J, Rajalingam K (2013). Endo-PDI is required for TNFα-induced angiogenesis. Free Radic Biol Med.

[74] Chang C, Rajasekaran M, Qiao Y, Dong H, Wang Y, Xia H (2022). The aberrant upregulation of exon 10-inclusive SREK1 through SRSF10 acts as an oncogenic driver in human hepatocellular carcinoma. Nat Commun.

[75] Zhang D, Richardson DR (2011). Endoplasmic reticulum protein 29 (ERp29): an emerging role in cancer. Int J Biochem Cell Biol.

[76] Gao H, Sun B, Fu H, Chi X, Wang F, Qi X (2016). PDIA6 promotes the proliferation of HeLa cells through activating the Wnt/β-catenin signaling pathway. Oncotarget.

[77] Torpe N, Gopal S, Baltaci O, Rella L, Handley A, Korswagen HC (2019). A protein disulfide isomerase controls neuronal migration through regulation of Wnt secretion. Cell Rep.

[78] Benham AM (2012). The protein disulfide isomerase family: key players in health and disease. Antioxidants Redox Signal.

[79] Willems SH, Tape CJ, Stanley PL, Taylor NA, Mills IG, Neal DE (2010). Thiol isomerases negatively regulate the cellular shedding activity of ADAM17. Biochem J.

[80] Campos JLO, Doratioto TR, Videira NB, Ribeiro Filho HV, Batista FAH, Fattori J (2018). Protein disulfide isomerase modulates the activation of thyroid hormone receptors. Front Endocrinol.

[81] Xu S, Sankar S, Neamati N (2014). Protein disulfide isomerase: a promising target for cancer therapy. Drug Discov Today.

[82] Jordan PA, Stevens JM, Hubbard GP, Barrett NE, Sage T, Authi KS (2005). A role for the thiol isomerase protein ERP5 in platelet function. Blood.

[83] Chien C-Y, Hung Y-J, Shieh Y-S, Hsieh C-H, Lu C-H, Lin F-H (2017). A novel potential biomarker for metabolic syndrome in Chinese adults: circulating protein disulfide isomerase family A, member 4. PLoS ONE.

[84] Thompson DA, Weigel RJ (1998). hAG-2, the human homologue of the *Xenopus*
*laevis* cement gland gene XAG-2, is coexpressed with estrogen receptor in breast cancer cell lines. Biochem Biophys Res Commun.

[85] Chevet E, Fessart D, Delom F, Mulot A, Vojtesek B, Hrstka R (2013). Emerging roles for the pro-oncogenic anterior gradient-2 in cancer development. Oncogene.

[86] Brychtova V, Mohtar A, Vojtesek B, Hupp TR (2015). Mechanisms of anterior gradient-2 regulation and function in cancer. Semin Cancer Biol.

[87] Alsereihi R, Schulten H-J, Bakhashab S, Saini K, Al-Hejin AM, Hussein D (2019). Leveraging the role of the metastatic associated protein anterior gradient homologue 2 in unfolded protein degradation: a novel therapeutic biomarker for cancer. Cancers.

[88] Tian S, Hu J, Tao K, Wang J, Chu Y, Li J (2018). Secreted AGR2 promotes invasion of colorectal cancer cells via Wnt11-mediated non-canonical Wnt signaling. Exp Cell Res.

[89] Tiemann K, Garri C, Lee SB, Malihi PD, Park M, Alvarez RM (2019). Loss of ER retention motif of AGR2 can impact mTORC signaling and promote cancer metastasis. Oncogene.

[90] Dong A, Wodziak D, Lowe AW (2015). Epidermal growth factor receptor (EGFR) signaling requires a specific endoplasmic reticulum thioredoxin for the post-translational control of receptor presentation to the cell surface. J Biol Chem.

[91] Park S-W, Zhen G, Verhaeghe C, Nakagami Y, Nguyenvu LT, Barczak AJ (2009). The protein disulfide isomerase AGR2 is essential for production of intestinal mucus. Proc Natl Acad Sci USA.

[92] Clarke DJ, Murray E, Faktor J, Mohtar A, Vojtesek B, MacKay CL (2016). Mass spectrometry analysis of the oxidation states of the pro-oncogenic protein anterior gradient-2 reveals covalent dimerization via an intermolecular disulphide bond. Biochim Biophys Acta Proteins Proteom.

[93] Dumartin L, Alrawashdeh W, Trabulo SM, Radon TP, Steiger K, Feakins RM (2017). ER stress protein AGR2 precedes and is involved in the regulation of pancreatic cancer initiation. Oncogene.

[94] Moidu NA, Rahman NSA, Syafruddin SE, Low TY, Mohtar MA (2020). Secretion of pro-oncogenic AGR2 protein in cancer. Heliyon.

[95] Maurel M, Obacz J, Avril T, Ding Y, Papadodima O, Treton X (2019). Control of anterior GRadient 2 (AGR2) dimerization links endoplasmic reticulum proteostasis to inflammation. EMBO Mol Med.

[96] Sicari D, Centonze FG, Pineau R, Le Reste P-J, Negroni L, Chat S (2021). Reflux of endoplasmic reticulum proteins to the cytosol inactivates tumor suppressors. EMBO Rep.

[97] Mohtar MA, Hernychova L, O’Neill JR, Lawrence ML, Murray E, Vojtesek B (2018). The sequence-specific peptide-binding activity of the protein sulfide isomerase AGR2 directs its stable binding to the oncogenic receptor EpCAM. Mol Cell Proteom.

[98] Delom F, Mohtar MA, Hupp T, Fessart D (2020). The anterior gradient-2 interactome. Am J Physiol Cell Physiol.

[99] Bouchalova P, Sommerova L, Potesil D, Martisova A, Lapcik P, Koci V (2022). Characterization of the AGR2 interactome uncovers new players of protein disulfide isomerase network in cancer cells. Mol Cell Proteom.

[100] Worfolk JC, Bell S, Simpson LD, Carne NA, Francis SL, Engelbertsen V (2019). Elucidation of the AGR2 interactome in esophageal adenocarcinoma cells identifies a redox-sensitive chaperone hub for the quality control of MUC-5AC. Antioxidants Redox Signal.

[101] Knoblach B, Keller BO, Groenendyk J, Aldred S, Zheng J, Lemire BD (2003). ERp19 and ERp46, new members of the thioredoxin family of endoplasmic reticulum proteins. Mol Cell Proteom.

[102] Ivanova AS, Tereshina MB, Ermakova GV, Belousov VV, Zaraisky AG (2013). Agr genes, missing in amniotes, are involved in the body appendages regeneration in frog tadpoles. Sci Rep.

[103] Alanen HI, Williamson RA, Howard MJ, Lappi A-K, Jäntti HP, Rautio SM (2003). Functional characterization of ERp18, a new endoplasmic reticulum-located thioredoxin superfamily member. J Biol Chem.

[104] Rowe ML, Ruddock LW, Kelly G, Schmidt JM, Williamson RA, Howard MJ (2009). Solution structure and dynamics of ERp18, a small endoplasmic reticulum resident oxidoreductase. Biochemistry.

[105] Jeong W, Lee D-Y, Park S, Rhee SG (2008). ERp16, an endoplasmic reticulum-resident thiol-disulfide oxidoreductase. J Biol Chem.

[106] Jessop CE, Watkins RH, Simmons JJ, Tasab M, Bulleid NJ (2009). Protein disulphide isomerase family members show distinct substrate specificity: P5 is targeted to BiP client proteins. J Cell Sci.

[107] Wu J, Chen X, Wang X, Yu Y, Ren J, Xiao Y (2015). ERp19 contributes to tumorigenicity in human gastric cancer by promoting cell growth, migration and invasion. Oncotarget.

[108] Adam PJ, Boyd R, Tyson KL, Fletcher GC, Stamps A, Hudson L (2003). Comprehensive proteomic analysis of breast cancer cell membranes reveals unique proteins with potential roles in clinical cancer. J Biol Chem.

[109] Liu D, Rudland PS, Sibson DR, Platt-Higgins A, Barraclough R (2005). Human homologue of cement gland protein, a novel metastasis inducer associated with breast carcinomas. Cancer Res.

[110] Salmans ML, Zhao F, Andersen B (2013). The estrogen-regulated anterior gradient 2 (AGR2) protein in breast cancer: a potential drug target and biomarker. Breast Cancer Res.

[111] Gray TA, MacLaine NJ, Michie CO, Bouchalova P, Murray E, Howie J (2012). Anterior Gradient-3: a novel biomarker for ovarian cancer that mediates cisplatin resistance in xenograft models. J Immunol Methods.

[112] King ER, Tung CS, Tsang YTM, Zu Z, Lok GTM, Deavers MT (2011). The anterior gradient homolog 3 (AGR3) gene is associated with differentiation and survival in ovarian cancer. Am J Surg Pathol.

[113] Garczyk S, von Stillfried S, Antonopoulos W, Hartmann A, Schrauder MG, Fasching PA (2015). AGR3 in breast cancer: prognostic impact and suitable serum-based biomarker for early cancer detection. PLoS ONE.

[114] Nguyen VD, Biterova E, Salin M, Wierenga RK, Ruddock LW (2018). Crystal structure of human anterior gradient protein 3. Acta Crystallogr F Struct Biol Commun.

[115] Hetz C (2012). The unfolded protein response: controlling cell fate decisions under ER stress and beyond. Nat Rev Mol Cell Biol.

[116] Hetz C, Chevet E, Oakes SA (2015). Proteostasis control by the unfolded protein response. Nat Cell Biol.

[117] Ron D, Walter P (2007). Signal integration in the endoplasmic reticulum unfolded protein response. Nat Rev Mol Cell Biol.

[118] Dejeans N, Manié S, Hetz C, Bard F, Hupp T, Agostinis P (2014). Addicted to secrete—novel concepts and targets in cancer therapy. Trends Mol Med.

[119] Yadav RK, Chae S-W, Kim H-R, Chae HJ (2014). Endoplasmic reticulum stress and cancer. J Cancer Prev.

[120] Chen X, Cubillos-Ruiz JR (2021). Endoplasmic reticulum stress signals in the tumour and its microenvironment. Nat Rev Cancer.

[121] Dejeans N, Barroso K, Fernandez-Zapico ME, Samali A, Chevet E (2015). Novel roles of the unfolded protein response in the control of tumor development and aggressiveness. Semin Cancer Biol.

[122] Hwang J, Qi L (2018). Quality control in the endoplasmic reticulum: crosstalk between ERAD and UPR pathways. Trends Biochem Sci.

[123] Oyadomari S, Mori M (2004). Roles of CHOP/GADD153 in endoplasmic reticulum stress. Cell Death Differ.

[124] Hetz C (2012). The unfolded protein response: controlling cell fate decisions under ER stress and beyond. Nat Rev Mol Cell Biol.

[125] Suwanmanee G, Yosudjai J, Phimsen S, Wongkham S, Jirawatnotai S, Kaewkong W (2020). Upregulation of AGR2vH facilitates cholangiocarcinoma cell survival under endoplasmic reticulum stress via the activation of the unfolded protein response pathway. Int J Mol Med.

[126] Zhang J, Yi M, Zha L, Chen S, Li Z, Li C (2016). Sodium butyrate induces endoplasmic reticulum stress and autophagy in colorectal cells: implications for apoptosis. PLoS ONE.

[127] Higa A, Mulot A, Delom F, Bouchecareilh M, Nguyên DT, Boismenu D (2011). Role of Pro-oncogenic protein disulfide isomerase (PDI) family member anterior gradient 2 (AGR2) in the control of endoplasmic reticulum homeostasis. J Biol Chem.

[128] Higa A, Taouji S, Lhomond S, Jensen D, Fernandez-Zapico ME, Simpson JC (2014). Endoplasmic reticulum stress-activated transcription factor ATF6α requires the disulfide isomerase PDIA5 to modulate chemoresistance. Mol Cell Biol.

[129] Kranz P, Neumann F, Wolf A, Classen F, Pompsch M, Ocklenburg T (2017). PDI is an essential redox-sensitive activator of PERK during the unfolded protein response (UPR). Cell Death Dis.

[130] Eletto D, Eletto D, Boyle S, Argon Y (2016). PDIA6 regulates insulin secretion by selectively inhibiting the RIDD activity of IRE1. FASEB J.

[131] Eletto D, Eletto D, Dersh D, Gidalevitz T, Argon Y (2014). Protein disulfide isomerase A6 controls the decay of IRE1α signaling via disulfide-dependent association. Mol Cell.

[132] Ataman-Onal Y, Beaulieu C, Busseret S, Charrier J-P, Choquet-Kastylevsky G, Rolland D. Protein disulfide isomerase assay method for the in vitro diagnosis of colorectal cancer. 2013. https://patents.google.com/patent/US8367806B2/en. Accessed 10 Nov 2021.

[133] Fonseca C, Soiffer R, Ho V, Vanneman M, Jinushi M, Ritz J (2009). Protein disulfide isomerases are antibody targets during immune-mediated tumor destruction. Blood.

[134] Darnell JE (2002). Transcription factors as targets for cancer therapy. Nat Rev Cancer.

[135] Hurst KE, Lawrence KA, Reyes Angeles L, Ye Z, Zhang J, Townsend DM (2019). Endoplasmic reticulum protein disulfide isomerase shapes T cell efficacy for adoptive cellular therapy of tumors. Cells.

[136] González-Santiago L, Alfonso P, Suárez Y, Núñez A, García-Fernández LF, Alvarez E (2007). Proteomic analysis of the resistance to aplidin in human cancer cells. J Proteome Res.

[137] Karala A-R, Ruddock LW (2010). Bacitracin is not a specific inhibitor of protein disulfide isomerase. FEBS J.

[138] Rosenberg N, Mor-Cohen R, Sheptovitsky VH, Romanenco O, Hess O, Lahav J (2019). Integrin-mediated cell adhesion requires extracellular disulfide exchange regulated by protein disulfide isomerase. Exp Cell Res.

[139] Xu S, Liu Y, Yang K, Wang H, Shergalis A, Kyani A (2019). Inhibition of protein disulfide isomerase in glioblastoma causes marked downregulation of DNA repair and DNA damage response genes. Theranostics.

[140] Mathys L, Balzarini J (2016). The role of cellular oxidoreductases in viral entry and virus infection-associated oxidative stress: potential therapeutic applications. Expert Opin Ther Targets.

[141] Klett D, Cahoreau C, Villeret M, Combarnous Y (2010). Effect of pharmaceutical potential endocrine disruptor compounds on protein disulfide isomerase reductase activity using di-eosin-oxidized-glutathion. PLOS ONE.

[142] Horibe T, Nagai H, Sakakibara K, Hagiwara Y, Kikuchi M (2001). Ribostamycin inhibits the chaperone activity of protein disulfide isomerase. Biochem Biophys Res Commun.

[143] Liu X-W, Sok D-E (2004). Inactivation of protein disulfide isomerase by alkylators including α, β-unsaturated aldehydes at low physiological pHs. Biol Chem.

[144] Descamps E, Petrault-Laprais M, Maurois P, Pages N, Bac P, Bordet R (2009). Experimental stroke protection induced by 4-hydroxybenzyl alcohol is cancelled by bacitracin. Neurosci Res.

[145] Godin B, Touitou E (2004). Mechanism of bacitracin permeation enhancement through the skin and cellular membranes from an ethosomal carrier. J Control Release.

[146] Jasuja R, Passam FH, Kennedy DR, Kim SH, van Hessem L, Lin L (2012). Protein disulfide isomerase inhibitors constitute a new class of antithrombotic agents. J Clin Investig.

[147] Lovat PE, Corazzari M, Armstrong JL, Martin S, Pagliarini V, Hill D (2008). Increasing melanoma cell death using inhibitors of protein disulfide isomerases to abrogate survival responses to endoplasmic reticulum stress. Cancer Res.

[148] Weston BS, Wahab NA, Roberts T, Mason RM (2001). Bacitracin inhibits fibronectin matrix assembly by mesangial cells in high glucose. Kidney Int.

[149] Dickerhof N, Kleffmann T, Jack R, McCormick S (2011). Bacitracin inhibits the reductive activity of protein disulfide isomerase by disulfide bond formation with free cysteines in the substrate-binding domain. FEBS J.

[150] Goplen D, Wang J, Enger P, Tysnes BB, Terzis AJA, Laerum OD (2006). Protein disulfide isomerase expression is related to the invasive properties of malignant glioma. Cancer Res.

[151] Campos SK, Chapman JA, Deymier MJ, Bronnimann MP, Ozbun MA (2012). Opposing effects of bacitracin on human papillomavirus type 16 infection: enhancement of binding and entry and inhibition of endosomal penetration. J Virol.

[152] Markovic I, Stantchev TS, Fields KH, Tiffany LJ, Tomiç M, Weiss CD (2004). Thiol/disulfide exchange is a prerequisite for CXCR4-tropic HIV-1 envelope-mediated T-cell fusion during viral entry. Blood.

[153] Ryser H, Levy EM, Mandel R, DiSciullo GJ (1994). Inhibition of human immunodeficiency virus infection by agents that interfere with thiol-disulfide interchange upon virus-receptor interaction. Proc Natl Acad Sci.

[154] Essex DW, Li M, Miller A, Feinman RD (2001). Protein disulfide isomerase and sulfhydryl-dependent pathways in platelet activation. Biochemistry.

[155] Lahav J, Wijnen EM, Hess O, Hamaia SW, Griffiths D, Makris M (2003). Enzymatically catalyzed disulfide exchange is required for platelet adhesion to collagen via integrin α2β1. Blood.

[156] Atkin JD, Farg MA, Turner BJ, Tomas D, Lysaght JA, Nunan J (2006). Induction of the unfolded protein response in familial amyotrophic lateral sclerosis and association of protein-disulfide isomerase with superoxide dismutase 1. J Biol Chem.

[157] Couët J, de Bernard S, Loosfelt H, Saunier B, Milgrom E, Misrahi M (1996). Cell surface protein disulfide-isomerase is involved in the shedding of human thyrotropin receptor ectodomain. Biochemistry.

[158] Higuchi T, Watanabe Y, Waga I (2004). Protein disulfide isomerase suppresses the transcriptional activity of NF-κB. Biochem Biophys Res Commun.

[159] Janiszewski M, Lopes LR, Carmo AO, Pedro MA, Brandes RP, Santos CX (2005). Regulation of NAD (P) H oxidase by associated protein disulfide isomerase in vascular smooth muscle cells. J Biol Chem.

[160] Mandel R, Ryser H, Ghani F, Wu M, Peak D (1993). Inhibition of a reductive function of the plasma membrane by bacitracin and antibodies against protein disulfide-isomerase. Proc Natl Acad Sci.

[161] Wajih N, Hutson SM, Wallin R (2007). Disulfide-dependent protein folding is linked to operation of the vitamin K cycle in the endoplasmic reticulum: a protein disulfide isomerase-VKORC1 redox enzyme complex appears to be responsible for vitamin K1 2, 3-epoxide reduction. J Biol Chem.

[162] Stojak M, Milczarek M, Kurpinska A, Suraj-Prazmowska J, Kaczara P, Wojnar-Lason K (2020). Protein disulphide isomerase A1 is involved in the regulation of breast cancer cell adhesion and transmigration via lung microvascular endothelial cells. Cancers.

[163] Xiong B, Jha V, Min J-K, Cho J (2020). Protein disulfide isomerase in cardiovascular disease. Exp Mol Med.

[164] Hoffstrom BG, Kaplan A, Letso R, Schmid RS, Turmel GJ, Lo DC (2010). Inhibitors of protein disulfide isomerase suppress apoptosis induced by misfolded proteins. Nat Chem Biol.

[165] Lara HH, Ixtepan-Turrent L, Garza-Treviño EN, Flores-Teviño SM, Borkow G, Rodriguez-Padilla C (2011). Antiviral propierties of 5, 5’-dithiobis-2-nitrobenzoic acid and bacitracin against T-tropic human immunodeficiency virus type 1. Virol J.

[166] Tsibris JC, Hunt L, Ballejo G, Barker W, Toney L, Spellacy W (1989). Selective inhibition of protein disulfide isomerase by estrogens. J Biol Chem.

[167] Khan MM, Simizu S, Lai NS, Kawatani M, Shimizu T, Osada H (2011). Discovery of a small molecule PDI inhibitor that inhibits reduction of HIV-1 envelope glycoprotein gp120. ACS Chem Biol.

[168] Xu S, Butkevich AN, Yamada R, Zhou Y, Debnath B, Duncan R (2012). Discovery of an orally active small-molecule irreversible inhibitor of protein disulfide isomerase for ovarian cancer treatment. Proc Natl Acad Sci USA.

[169] Bennett TA, Edwards BS, Sklar LA, Rogelj S (2000). Sulfhydryl regulation of L-selectin shedding: phenylarsine oxide promotes activation-independent L-selectin shedding from leukocytes. J Immunol.

[170] Gallina A, Hanley TM, Mandel R, Trahey M, Broder CC, Viglianti GA (2002). Inhibitors of protein-disulfide isomerase prevent cleavage of disulfide bonds in receptor-bound glycoprotein 120 and prevent HIV-1 entry. J Biol Chem.

[171] Gerhard R, John H, Aktories K, Just I (2003). Thiol-modifying phenylarsine oxide inhibits guanine nucleotide binding of Rho but not of Rac GTPases. Mol Pharmacol.

[172] MacRobbie EA (2002). Evidence for a role for protein tyrosine phosphatase in the control of ion release from the guard cell vacuole in stomatal closure. Proc Natl Acad Sci USA.

[173] Bekendam RH, Flaumenhaft R (2016). Inhibition of protein disulfide isomerase in thrombosis. Basic Clin Pharmacol Toxicol.

[174] Stopa JD, Neuberg D, Puligandla M, Furie B, Flaumenhaft R, Zwicker JI (2017). Protein disulfide isomerase inhibition blocks thrombin generation in humans by interfering with platelet factor V activation. JCI Insight.

[175] Zwicker JI, Schlechter BL, Stopa JD, Liebman HA, Aggarwal A, Puligandla M (2019). Targeting protein disulfide isomerase with the flavonoid isoquercetin to improve hypercoagulability in advanced cancer. JCI Insight.

[176] Banerjee R, Pace NJ, Brown DR, Weerapana E (2013). 1, 3, 5-Triazine as a modular scaffold for covalent inhibitors with streamlined target identification. J Am Chem Soc.

[177] Robinson RM, Reyes L, Duncan RM, Bian H, Reitz AB, Manevich Y (2019). Inhibitors of the protein disulfide isomerase family for the treatment of multiple myeloma. Leukemia.

[178] Hasipek M, Grabowski D, Guan Y, Alugubelli RR, Tiwari AD, Gu X (2021). Therapeutic targeting of protein disulfide isomerase PDIA1 in multiple myeloma. Cancers.

[179] Vatolin S, Phillips JG, Jha BK, Govindgari S, Hu J, Grabowski D (2016). Novel protein disulfide isomerase inhibitor with anticancer activity in multiple myeloma. Cancer Res.

[180] Wu Z-H, Zhu Q, Gao G-W, Zhou C-C, Li D-W (2010). Preparation, characterization and potential application of monoclonal antibody 18A4 against AGR2. Xi Bao Yu Fen Zi Mian Yi Xue Za Zhi.

[181] Guo H, Chen H, Zhu Q, Yu X, Rong R, Merugu SB (2016). A humanized monoclonal antibody targeting secreted anterior gradient 2 effectively inhibits the xenograft tumor growth. Biochem Biophys Res Commun.

[182] Negi H, Merugu SB, Mangukiya HB, Li Z, Zhou B, Sehar Q (2019). Anterior Gradient-2 monoclonal antibody inhibits lung cancer growth and metastasis by upregulating p53 pathway and without exerting any toxicological effects: a preclinical study. Cancer Lett.

[183] Mohtar MA, Low TY, Vojtesek B, Jamal R, Hupp T (2018). The development of a synthetic scFv monoclonal antibody targeting pro-oncogenic AGR2. Front Pharmacol.

[184] Venetianer P, Straub FB (1963). The enzymic reactivation of reduced ribonuclease. Biochim Biophys Acta.

[185] Freedman RB, Hirst TR, Tuite MF (1994). Protein disulphide isomerase: building bridges in protein folding. Trends Biochem Sci.

[186] Mikami R, Tsukagoshi S, Arai K (2021). Abnormal enhancement of protein disulfide isomerase-like activity of a cyclic diselenide conjugated with a basic amino acid by inserting a glycine spacer. Biology.

[187] Wang L, Yu J, Wang C (2020). Protein disulfide isomerase is regulated in multiple ways: consequences for conformation, activities, and pathophysiological functions. BioEssays..

[188] Lee E, Lee DH (2017). Emerging roles of protein disulfide isomerase in cancer. BMB Rep.

[189] Serrano A, Guyette JL, Heim JB, Taylor M, Cherubin P, Krengel U (2022). Holotoxin disassembly by protein disulfide isomerase is less efficient for *Escherichia*
*coli* heat-labile enterotoxin than cholera toxin. Sci Rep.

[190] Turano C, Coppari S, Altieri F, Ferraro A (2002). Proteins of the PDI family: unpredicted non-ER locations and functions. J Cell Physiol.

[191] Madina MH, Rahman MS, Huang X, Zhang Y, Zheng H, Germain H (2020). A poplar rust effector protein associates with protein disulfide isomerase and enhances plant susceptibility. Biology.

[192] Jumper J, Evans R, Pritzel A, Green T, Figurnov M, Ronneberger O (2021). Highly accurate protein structure prediction with AlphaFold. Nature.

